# Implementation and Evaluation of a Smartphone-Based Telemonitoring Program for Patients With Heart Failure: Mixed-Methods Study Protocol

**DOI:** 10.2196/resprot.9911

**Published:** 2018-05-03

**Authors:** Patrick Ware, Heather J Ross, Joseph A Cafazzo, Audrey Laporte, Emily Seto

**Affiliations:** ^1^ Institute of Health Policy, Management and Evaluation Dalla Lana School of Public Health University of Toronto Toronto, ON Canada; ^2^ Centre for Global eHealth Innovation Techna Institute University Health Network Toronto, ON Canada; ^3^ Ted Rogers Centre for Heart Research University Health Network Toronto, ON Canada; ^4^ Department of Medicine University of Toronto Toronto, ON Canada; ^5^ Peter Munk Cardiac Centre University Health Network Toronto, ON Canada; ^6^ Institute of Biomaterials and Biomedical Engineering University of Toronto Toronto, ON Canada; ^7^ Canadian Centre for Health Economics Toronto, ON Canada

**Keywords:** heart failure, telemedicine, self-management, health services research, costs and cost analysis

## Abstract

**Background:**

Meta-analyses of telemonitoring for patients with heart failure conclude that it can lower the utilization of health services and improve health outcomes compared with the standard of care. A smartphone-based telemonitoring program is being implemented as part of the standard of care at a specialty care clinic for patients with heart failure in Toronto, Canada.

**Objective:**

The objectives of this study are to (1) evaluate the impact of the telemonitoring program on health service utilization, patient health outcomes, and their ability to self-care; (2) identify the contextual barriers and facilitators of implementation at the physician, clinic, and institutional level; (3) describe patient usage patterns to determine adherence and other behaviors in the telemonitoring program; and (4) evaluate the costs associated with implementation of the telemonitoring program from the perspective of the health care system (ie, public payer), hospital, and patient.

**Methods:**

The evaluation will use a mixed-methods approach. The quantitative component will include a pragmatic pre- and posttest study design for the impact and cost analyses, which will make use of clinical data and questionnaires administered to at least 108 patients at baseline and 6 months. Furthermore, outcome data will be collected at 1, 12, and 24 months to explore the longitudinal impact of the program. In addition, quantitative data related to implementation outcomes and patient usage patterns of the telemonitoring system will be reported. The qualitative component involves an embedded single case study design to identify the contextual factors that influenced the implementation. The implementation evaluation will be completed using semistructured interviews with clinicians, and other program staff at baseline, 4 months, and 12 months after the program start date. Interviews conducted with patients will be triangulated with usage data to explain usage patterns and adherence to the system.

**Results:**

The telemonitoring program was launched in August 2016 and patient enrollment is ongoing.

**Conclusions:**

The methods described provide an example for conducting comprehensive evaluations of telemonitoring programs. The combination of impact, implementation, and cost evaluations will inform the quality improvement of the existing program and will yield insights into the sustainability of smartphone-based telemonitoring programs for patients with heart failure within a specialty care setting.

## Introduction

### Background

Currently, one of the greatest challenges for health care systems worldwide is the growing fiscal and social burden of preventing and managing chronic diseases [1]. Heart failure (HF) is one of the most expensive chronic diseases, partly because 50% of patients with HF get readmitted within 1 year [2]. Evidence suggests approximately half of all readmissions are preventable and result from inadequate discharge teaching, nonadherence to medication, or failure to have early follow-up with a clinician [3].

There is mounting evidence that embedding self-care within existing health care services provides an effective model to meet these needs for a number of chronic diseases, including HF [4-7]. Although more traditional self-care interventions such as health coaching or patient education can be effective [8,9], their implementation often proves challenging [10,11]. Health information technology is one avenue that can support the delivery of self-care interventions. For example, telemonitoring (TM) is thought to be crucial to offer patients the right care at the right time [12] by allowing patients to collect clinical data at home, which is then transmitted via technology to be viewed and acted upon by a clinician at a distant location [13].

In HF, meta-analyses conclude that TM lowers the utilization of health services, improves HF health outcomes, and improves health-related quality of life (QoL) [14-20]. However, results vary widely between individual trials [21], and 2 of the largest studies, the Tele-HF [22] and BEAT-HF trials [23], reported null results. One problem is that results come from trials of varying quality, and there is often little discussion about the intervention itself or the degree to which the patients have adhered to it over the course of the study [24]. Furthermore, studies looking at the implementation of TM have identified technical, cost, organizational, and behavioral barriers, which may explain why these technologies are not yielding consistent positive outcomes [12,25]. In addition, this may explain why, despite some evidence of cost-effectiveness [26], there is still inconclusive evidence regarding the impact of TM in terms of its cost to patients with HF [26].

Although the ubiquity of mobile phones is believed to have the potential to make TM interventions more accessible and cost-effective [27,28], additional knowledge gaps exist in the emerging use of smartphones for TM of chronic diseases. Randomized controlled trials (RCTs) employing mobile phone–based TM interventions have been conducted and have shown similar positive results to those reported for more traditional TM [29,30]. However, this type of TM is novel, and therefore, there have been no comprehensive implementation studies on smartphone-based TM interventions completed to date.

### Implementation of a Smartphone-Based Telemonitoring Program

#### The Intervention

An algorithm-based smartphone-based TM program for patients with HF, called the Medly Program, is being implemented as part of the standard of care at the University Health Network’s Ted Rogers Center of Excellence for Heart Function in Toronto, Canada (hereafter referred to as the HF clinic). The objective of the Medly Program is similar to other HF TM programs: improve patient self-management and decrease health care utilization [31]. Patients enrolled in the Medly Program will receive a Medly kit that includes a smartphone (Samsung Galaxy Grand Prime) with a limited data plan and the Medly app already downloaded. In addition, patients receive a Bluetooth-enabled weight scale and blood pressure cuff. Patients are instructed to take daily weight and blood pressure readings using these devices and record their symptoms using the Medly app first thing in the morning. Automated self-care instructions are immediately displayed in the Medly app after these 3 parameters are processed by an algorithm that was developed in close consultation with HF clinicians. In addition, patients have access to graphs displaying historical trends for these parameters. To assist in compliance, patients receive an automated call on their primary phone line if they have not taken their readings before 10 AM. The Medly Program is initially providing patients with all the equipment to mitigate the potential operational and software development challenges of offering the service on different devices during the critical early stages of implementation. However, a transition to a *bring your own device* model is planned, which would enable patients with smartphones to use their own devices by downloading the Medly app.

If there are signs of deteriorating health of a patient, the Medly algorithm generates an alert to a HF clinician who is part of the patient’s care team. The alerts are made available to clinicians in 2 formats. The first is through an automated email containing the latest weight, blood pressure, and symptoms along with the patient’s target ranges. In addition, the email contains the patients’ latest medication list and HF–related laboratory results, and contact information. Second, clinicians can choose to view the alerts by accessing a secure Web portal that presents a list of all the alerts triggered. Here, clinicians can review details of the latest alert and graphs showing historical weight, blood pressure, symptoms, and HF–specific laboratory results, which are visually contextualized according to the patient’s target ranges. The clinician will follow-up with the patient depending on the clinical need, documenting all actions and decisions taken in response to the alert in the hospital electronic medical record (EMR).

The Medly Program is intended to be delivered as part of the standard of care; as such, the goal is for it to be seamlessly integrated within the existing workflows of the HF clinic. Before deployment, a service blueprint was created through ethnographic methods including observation and informal interviews with clinicians and support staff in the HF clinic. This helped identify areas where the processes of the Medly Program could be incorporated. In addition, an RCT of 100 patients with an embedded qualitative component was previously conducted in the HF clinic with an earlier version of the Medly system. This study concluded that patients receiving the intervention experienced improvements in QoL and self-care maintenance compared with a control group [30]. Lessons learned from this RCT helped justify the decision to implement the Medly Program as part of the standard of care and informed the program’s implementation strategy. The current program offers an opportunity to evaluate the implementation and effectiveness of Medly under real-world conditions.

#### User Training and Support

Training as to how to use the Medly app and associated devices is provided to patients at the time of enrollment into the Medly Program by a telehealth analyst. Clinicians monitoring patients through the Medly system participated in a formal training session approximately 1 month before program deployment. Both patients and clinicians are provided with a user manual to supplement the in-person training along with contact information of the telehealth analyst who offers technical support during normal business hours.

### Evaluation Objectives and Research Questions

#### Objective 1

The first objective is to evaluate the impact of the Medly Program on health service utilization, patient health outcomes, and their ability to self-care. Alerts sent to patients and clinicians will help identify periods of symptom exacerbation and volume overload. The impact of this is expected to permit earlier intervention for worsening conditions, thus avoiding trips to the emergency department (ED) and hospitalizations. For patients who are enrolled into the Medly Program on hospital discharge, the program is expected to reduce 30-day readmission rates. In addition, participation in the Medly Program is expected to improve patients’ ability to self-care, leading to improved clinical outcomes and QoL.

#### Objective 2

The second objective is to evaluate the degree to which the Medly Program was implemented as intended and to identify the contextual barriers and facilitators of implementation. This objective will answer the following questions:

- To what extent did the HF clinic implement the Medly Program as intended?
- What contextual factors influence the implementation of the Medly Program?
- What adaptations were needed to implement and sustain the program within existing clinical workflows?

#### Objective 3

The third objective is to describe patient usage patterns to determine adherence and other behaviors in the Medly Program. This objective will answer the following questions:

- To what degree do patients adhere to the Medly Program and how do adherence patterns change over time?
- What factors influence patient adherence?

#### Objective 4

The fourth objective is to evaluate the costs associated with the implementation of the TM program from the perspective of the health care system (ie, public payer), the hospital, and patients.

## Methods

### Overview of the Study Design and Evaluation Framework

Data for the 4 objectives will be collected using mixed-methods. This approach will include a multiple pre-and posttest design for the evaluation of patient-level impacts, patient adherence, and cost. Quantitative data will include data collected as part of the standard of care (including health care utilization data and laboratory results obtained using a chart review) and usage data from the TM system. Additional patient-level data will be collected using questionnaires at baseline, 1 month, 6 months, 12 months, and 24 months. The qualitative component will take the form of an embedded single case study [32]. The 2 embedded subunits of analysis include clinicians and patients, as it relates to their adoption and use of the Medly system. The case is defined as the Medly Program at the HF clinic for the duration of 1 year starting from the program’s launch date (August 23, 2016). Qualitative methods, including semistructured interviews and document reviews, will be used to gain insights regarding patient self-care practices (objective 1), the barriers and facilitators to program implementation (objective 2), and explanations for patient adherence and usage of the system (objective 3).

### Study Participants

Representatives from all stakeholder groups involved in the implementation of the Medly Program will be recruited for participation.

#### Patients

Patients can be enrolled into the Medly Program provided they (1) are 18 years or older, (2) have been diagnosed with HF and are followed by a cardiologist at the HF clinic, (3) can speak and read English (or have an informal caregiver who does) to adequately understand the text prompts in the Medly app, and (4) are able to comply with using Medly (eg, able to stand on the weight scale, able to answer symptom questions). As the Medly program is being implemented as part of the standard of care, there is no explicit exclusion criteria for participating in the program and its evaluation. The duration of program participation will be decided by patients and their treating cardiologist on an individual basis.

Upon enrollment into the Medly Program, patients will be presented with the option of answering questionnaires and participating in interviews related to the program evaluation. Patients will be asked to sign a written consent form before participating in the evaluation.

#### Program Staff

All members of the Medly Program staff will be asked to participate in semistructured interviews. These include clinicians providing care for patients with HF in the HF clinic (n=7), telehealth analyst (n=1), project manager (n=1), and members of the implementation team (n=2). These individuals will be asked to sign a consent form before their first interview.

### Data Collection and Analysis

#### Objective 1: Measuring the Impact of the Medly Program

##### Impact Indicators

The primary outcome for evaluating the impact of the Medly Program is the number of hospitalizations because of HF in the 6 months before versus the 6 months after enrollment. Secondary impact outcomes comparing 6-month to baseline values are described below.

###### Health Service Utilization

The number of hospitalizations (all-cause), 30-day readmission rate, days in hospital (HF and all-cause), number of ED visits (HF and all-cause), visits to family doctor (HF and all-cause), number of HF–related outpatient visits, and changes to medication will be recorded.

###### Left Ventricular Fraction and Laboratory Tests

The following HF–specific clinical parameters will be collected: left ventricular ejection fraction (LVEF), brain natriuretic peptide (BNP), creatinine, sodium, potassium, hemoglobin, and uric acid levels.

###### Mortality and Prediction of Survival

Patient mortality will be tracked. In addition, the Seattle Heart Failure Model (SHFM) will be calculated at program entry. Projected SHFM survival versus actual survival will be compared [33]. The calculation of this score requires data on age, gender, New York Heart Association classification, weight, LVEF, systolic blood pressure, list of medications (including diuretics), laboratory results (hemoglobin, lymphocytes, uric acid, total cholesterol, and sodium), and QRS interval.

###### Dyspnea

Patients will be asked to describe their level of breathlessness using a visual analogue scale for dyspnea on a scale ranging from 0 (no shortness of breath) to 10 (shortness of breath is the worst it can be).

###### Quality of Life

The EQ-5D-5L is a measure of generic health status and will be administered to all patients as a measure of QoL [34,35]. This 5-item instrument has undergone validity and reliability testing for several conditions including HF. Due to the generic nature of this measure, it is recommended that it be administered along with supplementary measurements to capture more disease-specific aspects related to QoL. Hence, the Minnesota Living with Heart Failure Questionnaire (MLHFQ) will also be administered. The questionnaire contains 21 items scored on a 5-point Likert scale, the responses of which are summed to produce a total score and subscores for the domains of physical and emotional QoL. The MLHFQ is widely used in studies involving HF–related QoL and has been shown to have a high level of reliability and validity [36].

###### Self-Care

The self-care of HF index asks respondents to respond to 22 items on a 4-point Likert scale to assess their ability to self-care across 3 subscales (maintenance, management, and confidence). The tool has undergone validity and reliability testing involving patients with HF [37].

###### Demographic Variables

Demographic information will also be collected using a questionnaire, which includes questions on the following: age, sex, income, native language, living arrangements (living alone, with a partner, or other), whether or not they have a caregiver (formal or informal), and type of living areas (eg, urban, suburban, or rural). In addition, patient comorbidities will be tracked. Finally, questions will be asked to assess the patient’s experience and comfort level with technology and smartphones, including frequency of use.

##### Data Acquisition

Data collection will occur at baseline, 1 month, 6 months, 12 months, and 24 months, or until the patient exits the program. Data collected in addition to the primary evaluation period (baseline to 6 months) will provide an opportunity for post-hoc analyses aimed at quality improvement, including recommended duration of use. For example, the 1-month time point was included to determine if the bulk of changes to self-care and QoL occur immediately after enrollment. Similarly, longitudinal data will help determine whether patient-level impacts are sustained or change when using the system long term.

Health service utilization, laboratory results, mortality, prediction of survival, and select demographic information will be obtained from the hospital EMR. In addition, health service utilization information will be verified by patient participants through self-reports via a questionnaire.

Baseline and follow-up questionnaires containing the validated survey tools listed in Table 1 will be distributed to patients during regularly scheduled visits. Upon study enrollment, patients will be asked whether they prefer to be mailed the questionnaire or completing it using an online survey tool (SurveyMonkey [38]) in the event that they do not have a clinic visit scheduled for the data collection time point. In these situations, the questionnaires will be sent to the patient according to the preferred format and they will be given 2 weeks to respond, after which a member of the evaluation team will call the patient to remind them to complete the questionnaire.

**Table 1:** Timing of outcome assessments for the impact evaluation.

Domain and measure		Baseline	1 month	6 months	12 months	24 months	Exit
**Health service utilization**							
	30-day readmission		X^a^				
	Number of hospitalizations	X		X	X	X	X
	Number of days in hospital	X		X	X	X	X
	Number of emergency department visits	X		X	X	X	X
	Number of heart failure-related outpatient visits	X		X	X	X	X
	Number of visits to family doctors	X		X	X	X	X
	Changes to medication	X		X	X	X	X
**Clinical outcomes**							
	Left ventricular ejection fraction	X			X	X	
	Blood work: BNP^b^, creatinine, sodium, potassium, hemoglobin, and uric acid	X		X	X	X	X
	Visual analogue scale for dyspnea	X	X	X	X	X	X
	SHFM^c^	X					
**Self-care**							
	SCHFI^d^ [37]	X	X	X	X	X	X
	EQ-5D-5L^e^ [34,35]	X	X	X	X	X	X
	MLHFQ^f^ [36]	X	X	X	X	X	X

^a^X: data is collected at this time point.

^b^BNP: brain natriuretic peptide.

^c^SHFM: Seattle Heart Failure Model.

^d^SCHFI: Self-Care of Heart Failure Index.

^e^EQ-5D-5L^:^ EuroQol five-dimensional.

^f^MLHFQ: Minnesota Living with Heart Failure Questionnaire.

##### Planned Analyses

The primary analyses will be paired Student *t* tests and Wilcoxon signed rank tests comparing baseline and 6-month values for all patient-level outcomes. For patients enrolled in the Medly Program on hospital discharge, the 30-day readmission rate will be compared with the readmission rate before the launch of the Medly Program, as determined using hospital administrative data. Secondary analyses aimed at determining the longitudinal impact of the Medly Program (ie, using outcome data from the additional time points), and the correlation of independent variables (eg, patient characteristics and adherence rates) with outcomes will be analyzed using general linear mixed model procedures. In addition, descriptive statistics will be produced for all variables collected, which may inform necessary subgroup analyses. All statistical analyses will be performed using the statistical software application SPSS (IBM Corporation, USA) [39].

##### Power Consideration

We will aim to recruit at least 108 patients into the Medly Program at the HF clinic before analyses of patient-level outcomes are undertaken. This number is based on being able to detect a small effect size (Cohen *d*=0.3) in the number of hospitalizations because of HF within the first 6 months of enrollment with 80% power and an alpha of .05 (two-sided) [40]. This number considers that approximately 20% of patients will be “lost to follow-up,” which includes patient mortality and those who withdraw from the program before the 6-month time point. We anticipate recruiting this number in the first 18 months of the program.

#### Objective 2: Implementation Evaluation

The mixed-methods implementation evaluation will be guided by the framework by Proctor et al, which describes outcomes that can serve as indicators of implementation success [41], and the Consolidated Framework for Implementation Research (CFIR). The CFIR describes factors influencing implementation success according to 5 domains: (1) intervention characteristics, (2) outer setting, (3) inner setting, (4) characteristics of individuals, and (5) process [42].

**Table 2:** Implementation outcome indicators.

Implementation outcome	Definitions^a^	Indicator
Adoption	The intention, initial decision, or action to try or employ an innovation or evidence-based practice. Adoption may also be referred to as “uptake”	Number of clinicians having decided to use Medly to monitor patients
Implementation cost	The cost impact of the implementation effort	See objective 4 for cost outcomes associated with implementation of the Medly Program
Feasibility	The extent to which a new treatment, or an innovation, can be successfully used or carried out within a given agency or setting	Number of patients enrolled; rate of patient enrollment; number of patient-initiated dropouts from the program; number of physician-initiated dropouts from the program—or no uptake
Fidelity	The degree to which an intervention was implemented as it was prescribed in the original protocol or as it was intended by the program developers	Number and nature of calls or emails made to the telehealth analyst; proportion of the number of—alerts acknowledged over the total number of alerts, phone calls to patients over the total number of alerts triggered, and false or inappropriate alerts over the total alerts triggered
Penetration	The integration of a practice within a service setting and its subsystems	Percentage of clinicians using Medly over the total number of potential clinician users in the HF clinic

^a^Definitions are based on the definitions provided by Proctor et al [41].

##### Implementation Outcome Indicators

Implementation outcomes are defined by Proctor et al as “the effects of deliberate and purposive actions to implement new treatments, practice, and services” [41]. A total of 5 implementation outcomes were selected as quantitative indicators and are presented in Table 2. These data will be obtained from the hospital’s EMR, the Medly system’s audit trails, and technical support logs.

##### Semistructured Interviews With Program Staff

Separate semistructured interview guides based on the constructs in the CFIR will be formulated for each category of program staff previously outlined. Interviews will be conducted at baseline, 4 months, and 12 months to align with the different phases of Stetler et al’s typology of formative evaluations [43]. The interviews are expected to last approximately 30 min and will be conducted at a location convenient to the participants. All interviews will be audiotaped for later transcription and analysis.

##### Planned Analyses

Descriptive statistics will be produced for the indicators of the implementation outcome to provide an objective measure of implementation success.

Furthermore, 2 independent researchers will analyze interview transcripts and documents using the Framework Method of qualitative analysis [44]. An initial round of coding will use a deductive approach by looking for themes that match the constructs in the CFIR. A second round of coding will be inductive using an open coding approach, which will involve researchers looking for unexpected themes that are not represented in the guiding CFIR framework. Throughout the analysis, both reviewers will discuss the themes and codes from their independent analyses to come up with a single analytical framework. This finalized framework will be applied in a final coding of all transcriptions using NVivo (QSR International, Doncaster, Victoria, Australia) [45].

Quantitative and qualitative data will be triangulated such that the interview data will help explain success or failure of the implementation.

#### Objective 3: Describing Patient Adherence and Usage Patterns

A sequential explanatory mixed-methods approach [46] will be used consisting of a quantitative measurement of patient adherence and semistructured interviews with patients at 4 time points.

##### Patient Adherence

Adherence will be assessed by analyzing patient usage rates of the Medly system—specifically, the proportion of days for which the patient took a complete reading (weight, blood pressure, and symptoms) over the previous 30 days. Usage data will be obtained and exported on a regular basis through Google Analytics [47].

##### Semistructured Interviews

A sample of patients enrolled in the Medly Program will participate in semistructured interviews aimed at understanding reasons for adherence or nonadherence and their general experiences with the Medly Program. Interview guides will be formulated based on the constructs of the Unified Theory of Acceptance and Use of Technology 2 [48]. For example, we will explore how patients’ expectations, ease of use of the intervention, facilitating conditions (eg, quality of technical support services), and the direct or indirect influence of clinicians and loved ones could explain levels of uptake, adherence, and use. Unlike adherence, which will be calculated for all patients enrolled in the Medly Program, interviews will be conducted until information saturation is reached using maximum variation sampling [49] based on age, sex, experience with technology, health status, time since enrollment, and level of adherence. As recommended by Francis et al for theory-based interview studies, an a priori target of 10 patients is being set as the initial analysis sample [50]; however, interviews will continue until no new themes emerge [50]. For patients who withdraw from the Medly Program before the end of the evaluation period, reasons for this withdrawal will be documented as part of standardized off-boarding procedures and a sample will be asked to participate in an interview. The interviews are expected to last 20 to 30 min and will be conducted in a quiet and private space within the clinic (eg, consultation room) during a regular clinic visit or over the telephone. All interviews will be audiotaped and transcribed for later analysis.

##### Planned Analyses

Monthly adherence rates will be examined using descriptive statistics to identify any patterns in the increase or decrease of patient adherence to Medly over time. In addition, general linear mixed model procedures will be performed to determine if any baseline patient characteristics (eg, age, sex, and HF severity) or time since program enrollment predicts patient adherence to the Medly system. Semistructured interviews will be analyzed using the Framework Method [44], as previously described.

#### Objective 4: Cost Impact of Implementing the Medly Program

The costs associated with implementing the Medly Program will be determined from the perspectives of the public payer, hospital, and patient. In reporting these results, costs will be interpreted in relation to the patient-level impacts determined in objective 1.

##### Data Acquisition

Costs will be calculated using a 6-month time frame. Specifically, we will compare costs before the implementation of the Medly Program (assessed at baseline) versus the costs after enrollment of patients into the program (measured at 6 months). This time frame was chosen because it represents the time horizon over which most of the health effects and costs of using the Medly Program are expected. Most of the cost variables will be self-reported by patients and triangulated using administrative data whenever possible (eg, EMR data). Therefore, questions related to the cost will be added to the patient outcome questionnaires (objective 1) and will be administered at baseline and 6 months.

###### Public Payer Perspective

Costs to the public payer will be determined by looking at health care utilization of patients enrolled in the Medly Program before versus after their enrollment. These will include hospitalizations, ED visits, HF clinic visits, family physician visits, and use of home care services. In addition, costs for inpatient medications will be considered.

###### Hospital Perspective

Costs from the hospital perspective will be valued based on time spent by human resources involved in the Medly Program. This time will be converted to costs based on those individuals’ respective salaries. This will include time the clinicians spent reviewing and responding to Medly alerts as well as time the clinicians spend in training sessions, learning to use the system, and seeking technical support. In addition, costs for the hospital perspective will include equipment costs (Medly kit, smartphone data plan, and server) and the salary for employing a telehealth analyst responsible for recruiting, training, onboarding, managing inventory support, and providing training.

###### Patient Perspective

Costs for patients will primarily be determined by the time they spend accessing care for HF. This will involve determining their employment status and annual income as well as time they spend traveling to and from appointments, time spent at appointments, and how much work time was missed because of their HF condition (vacation or unpaid). In addition, this will include the time patients spent learning to use the Medly system, time spent using the system, and time getting technical support. Additional costs considered include travel, parking, and all other out-of-pocket costs related to accessing care or using the intervention. Moreover, time of informal caregivers (eg, friend or family member who helps the patient to manage their HF) will be valued as part of the patient perspective. Costs for informal caregivers will be based on the average hourly rate of personal support workers.

## Results

The Medly Program was launched in August 2016. As of April 4, 2018, 166 patients have been enrolled. The primary impact analysis is expected to be conducted by January 2019.

## Discussion

This study aims to evaluate the implementation and impact of a smartphone-based TM program being implemented as part of the standard of care in a specialty care setting in a large Canadian city.

### Limitations

Unlike TM systems evaluated in the context of academic research, the lack of strict patient inclusion and exclusion criteria for the Medly Program has the potential to lead to heterogeneity among evaluation participants, which will make it difficult to generalize the results to other health care settings. Another important limitation is that the nature of this evaluation and the availability of data do not allow for a distinct comparator group. Without discounting these limitations, we believe that one of the strengths of this evaluation is its pragmatic nature and that these threats to internal and external validity will be mitigated through a detailed description of the context and participants when results are reported. The interpretation of the Medly Program evaluation results will include comparisons with previous TM RCTs conducted within the HF clinic [30] and other comparable settings, as well as other RCTs evaluating interventions designed to promote self-care through education and health coaching conducted within the HF clinic [51]. This is possible because of the selection of outcome metrics common in other TM studies.

### Conclusions

Unlike other TM studies that focus primarily on quantitative outcomes, this evaluation will also examine the context and mechanisms that lead to them. Therefore, this pragmatic mixed-methods study will allow for an interpretation of results using realist evaluation principles [52]. The information gathered during this evaluation will inform if, and how, a smartphone-based TM system improves the self-care capacities, clinical management, and health outcomes of patients with HF. This evaluation will lead to quality improvement of the current program and provide evidence that will inform the implementation and sustainability of other TM programs.

## Abbreviations

BNP: brain natriuretic peptide
CFIR: Consolidated Framework for Implementation Research
ED: emergency department
EMR: electronic medical record
EQ-5D-5L: EuroQol five-dimensional
HF: heart failure
LVEF: left ventricular ejection fraction
MLHFQ: Minnesota Living with Heart Failure Questionnaire
QoL: quality of life
RCTs: randomized controlled trials
SCHFI: Self-Care of Heart Failure Index
SHFM: Seattle Heart Failure Model
TM: telemonitoring
